# Effect of Phenylephrine on the Accommodative System

**DOI:** 10.1155/2016/7968918

**Published:** 2016-12-07

**Authors:** José J. Esteve-Taboada, Antonio J. Del Águila-Carrasco, Paula Bernal-Molina, Teresa Ferrer-Blasco, Norberto López-Gil, Robert Montés-Micó

**Affiliations:** ^1^Department of Optics and Optometry and Visual Sciences, University of Valencia, Valencia, Spain; ^2^Mixed group UVEG-UMU, Interuniversity Laboratory for Research in Vision and Optometry, Valencia, Spain; ^3^Vision Sciences Research Group (CiViUM), University of Murcia, Murcia, Spain

## Abstract

Accommodation is controlled by the action of the ciliary muscle and mediated primarily by parasympathetic input through postganglionic fibers that originate from neurons in the ciliary and pterygopalatine ganglia. During accommodation the pupil constricts to increase the depth of focus of the eye and improve retinal image quality. Researchers have traditionally faced the challenge of measuring the accommodative properties of the eye through a small pupil and thus have relied on pharmacological agents to dilate the pupil. Achieving pupil dilation (mydriasis) without affecting the accommodative ability of the eye (cycloplegia) could be useful in many clinical and research contexts. Phenylephrine hydrochloride (PHCl) is a sympathomimetic agent that is used clinically to dilate the pupil. Nevertheless, first investigations suggested some loss of functional accommodation in the human eye after PHCl instillation. Subsequent studies, based on different measurement procedures, obtained contradictory conclusions, causing therefore an unexpected controversy that has been spread almost to the present days. This manuscript reviews and summarizes the main research studies that have been performed to analyze the effect of PHCl on the accommodative system and provides clear conclusions that could help clinicians know the real effects of PHCl on the accommodative system of the human eye.

## 1. Introduction

Accommodation is the change in optical power experienced by the crystalline lens when the ciliary muscle contracts, which allows the human eye to focus on near objects. The eye uses this mechanism to attain clear vision across a wide range of viewing distances. In an emmetropic eye, accommodation is relaxed when the eye is focusing at distant objects. This system is quite precise and its operation depends on the integrity of both central and peripheral connections. Accommodative dysfunction, which may be either excessive or insufficient, can be caused by both systemic abnormalities and focal pathologic processes.

Accommodation is controlled by the action of the ciliary muscle, which is innervated by the autonomic nervous system (ANS). This system influences numerous ocular functions through parasympathetic and sympathetic innervations. The control of accommodation is mediated primarily by parasympathetic input [1], resulting in changes in the dioptric power of the crystalline lens. Nevertheless, there exists evidence supporting also a sympathetic innervation of ciliary muscle [2].

Parasympathetic innervation is mediated through postganglionic fibers that originate from neurons in the ciliary and pterygopalatine ganglia [3]. Ciliary ganglion neurons project to the ciliary body and the iris sphincter muscle to control accommodation and pupil constriction, respectively. Pupil dilation is controlled by the iris dilator muscle through sympathetic innervation from postganglionic fibers, which have their origin in neurons at the superior cervical ganglion. During accommodation the pupil constricts to increase the depth of focus of the eye and improve retinal image quality [4]. This is part of the accommodation reflex, which includes all the automatic and coordinated changes that occur in the eye when viewing a near object: constriction of the pupil, convergence of the eyes, and increased convexity of the crystalline lens.

As a consequence of the accommodation reflex, researchers have traditionally faced the challenge of measuring the accommodative properties of the eye through a small pupil and thus have relied on pharmacological agents to dilate the pupil. Achieving pupil dilation (mydriasis) without affecting the accommodative ability of the eye (cycloplegia) could be useful in many clinical and research contexts.

Phenylephrine hydrochloride (PHCl) is a sympathomimetic agent that is used clinically to dilate the pupil in studies of accommodation, improving the ophthalmic instruments' performance when measuring through dilated pupils. Nevertheless, investigations in humans have suggested some loss of functional accommodation after PHCl instillation [5], although there exists some controversy whether this loss is due to direct action of PHCl on the ciliary muscle or due to secondary optical factors associated with mydriasis.

To date, multiple researchers have used PHCl to achieve mydriasis in human studies of accommodation without a clear understanding of its effect on the ciliary muscle. The aim of this manuscript is to review and summarize the main research studies that have been performed to analyze the effect of PHCl on the accommodative system and try to provide clear conclusions on this area that could help clinicians know the real effects of PHCl on the accommodative system of the human eye.

## 2. Methods of Literature Search

Review of the literature was conducted by searching in all these databases: PubMed (US National Library of Medicine), Web of Science (Thomson Reuters), Embase (Reed Elsevier Properties SA), and Scopus (Elsevier BV). We limited our search to English publications and peer reviewed scientific reports. No date restriction was used in the electronic searches. The date of the last electronic search was July 20, 2016.

In our literature search, we included a combination of keywords, defining the following search criteria: “accommodation” AND “phenylephrine OR PHCl”; we also screened the reference lists of all eligible studies to ensure that any relevant studies were not omitted.

After thoroughly searching the literature using these criteria, a total number of 15 peer reviewed relevant articles were analyzed. Of these 15 articles, 12 were research reports using different subjective and objective measurement procedures, 1 was reporting an in vitro pharmacological test using human ciliary muscle tissue, 1 was devoted to the study of accommodation responses (AR) in anesthetized rhesus monkeys, as they represent a proper model for human accommodation, since they have an accommodative mechanism and anatomy similar to that of humans, and the last one was measuring human ciliary muscle size in vivo using Optical Coherence Tomography (OCT) images. The oldest peer reviewed manuscript is dated 1959, while the more recent one is from 2016.

## 3. Autonomic Control of Accommodation

**Figure 1:**
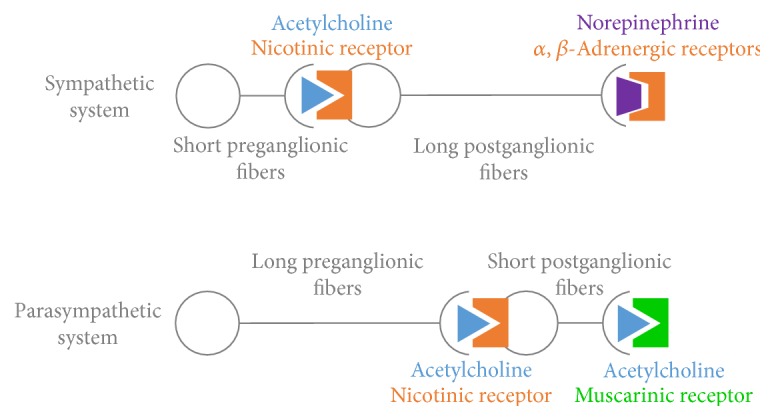
Schematic diagram of both sympathetic and parasympathetic divisions from the autonomic nervous system, showing the main receptors and neurotransmitters involved in each case.

The human body is equipped with a special branch of the nervous system called the ANS, which automatically controls and regulates the internal organs without any conscious recognition by the organism. The ANS is divided into two separate systems: the parasympathetic and the sympathetic nervous system. The sympathetic nervous system is involved in the stimulation of activities that prepare the body for action, while the parasympathetic nervous system activates tranquil functions. Sympathetic and parasympathetic divisions typically work in opposition to each other. This natural opposition is better understood as complementary in nature rather than antagonistic.  Figure 1 shows a schematic diagram of both sympathetic and parasympathetic divisions, showing the main receptors and neurotransmitters involved in each one.

Autonomic control of accommodation is predominantly parasympathetic and mediated by the action of acetylcholine on muscarinic receptors [19]. Parasympathetic input to the ciliary muscle mediates positive accommodation and meets the need for rapid focusing changes because of its fast onset of action (1-2 s). There is, however, clear evidence for a small inhibitory sympathetic input [20] associated with the parasympathetic response. Von Graefe, in 1855, was the first to suggest that the ciliary muscle must have a dual innervation by both parasympathetic and sympathetic nerves, as do the iris [21] and the great majority of autonomically innervated structures. Anatomic [22, 23], physiological [24, 25], and pharmacologic [12, 26, 27] signs have later demonstrated this hypothesis. In effect, anatomical and physiological literature provides compelling evidence that sympathetic innervation of human ciliary muscle produces inhibition of accommodation that is relatively small and slow and a function of the concurrent level of parasympathetic activity [20].

Sympathetic innervation to the ciliary muscle is mediated by the action of noradrenaline on two subclasses of postsynaptic receptors, *α*- and *β*-adrenoceptors. The stimulation of these receptors, which are located on the cell surface, produces different physiological effects. Using adrenergic blocking agents, Kern and posteriorly van Alphen demonstrated that human ciliary muscle was predominantly populated by *β*-adrenoceptors [19].

Pharmacological and nerve stimulation studies on animal models (monkeys) suggest that the sympathetic input is more relevant to tasks requiring sustained accommodation rather than to tasks requiring a rapid change in the AR. The effects of sympathetic inputs are small (less than 2 diopters (D)) and slow (10–40 s) and the magnitude of sympathetic inhibitory effects is related to the level of concurrent background parasympathetic activity [28].

As Gilmartin et al. proposed [29], the role of sympathetic innervation of the ciliary muscle may, for example, be to attenuate the retention of accommodative tone induced by periods of intense close work and thus reduce the risk of latent posttask transitory pseudomyopic changes. The principal characteristics of sympathetic inhibition were demonstrated in their study: it is inhibitory and mediated by *β*-adrenoceptors, is relatively small in magnitude, requires sustained augmentation of concurrent accommodative activity to become manifest, and operates in only a proportion of individuals. In particular, and as an example, 25–35% of young adult subjects have access to sympathetic inhibitory accommodative control [30, 31]. Absence of sympathetic facility has no significant effect on the function of the accommodative system under closed-loop visual stimuli. Besides, under high-contrast closed-loop conditions, the sympathetic branch of accommodative control fails to have a significant effect on manifest oculomotor responses [30].

However, it is important to highlight that when interpreting results of studies involving agents from the ANS, the complexity of the autonomic structures and their functions, as well as the potential interactions between them, should be taken into account [19].

## 4. Phenylephrine Hydrochloride (PHCl)

**Figure 2:**
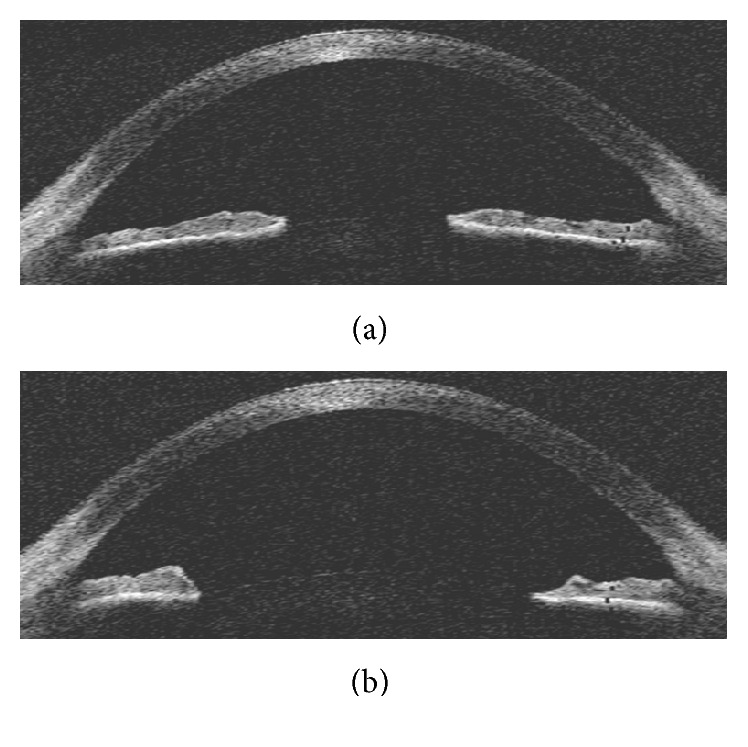
Anterior segment image of the human eye taken by Optical Coherence Tomography by means of the Visante Omni System (Carl Zeiss AG, Oberkochen, Germany). (a) Under myosis conditions (constriction of the pupil). (b) Under mydriatic conditions (dilation of the pupil).

PHCl is a selective *α*-adrenergic receptor (or *α*-adrenoceptor) agonist used primarily as a decongestant, as an agent to dilate the pupil, and to increase blood pressure. It is used as a mydriatic agent, in the form of eye drops, to dilate the iris before eye surgery or eye examinations. PHCl 2.5% is normally used for fundus examination, while 10% concentration is used therapeutically to break posterior synechiae and pupillary block [5].  Figure 2 shows two OCT anterior segment images of the human eye taken with the Visante Omni System (Carl Zeiss AG, Oberkochen, Germany) under myosis and mydriasis.

**Figure 3:**
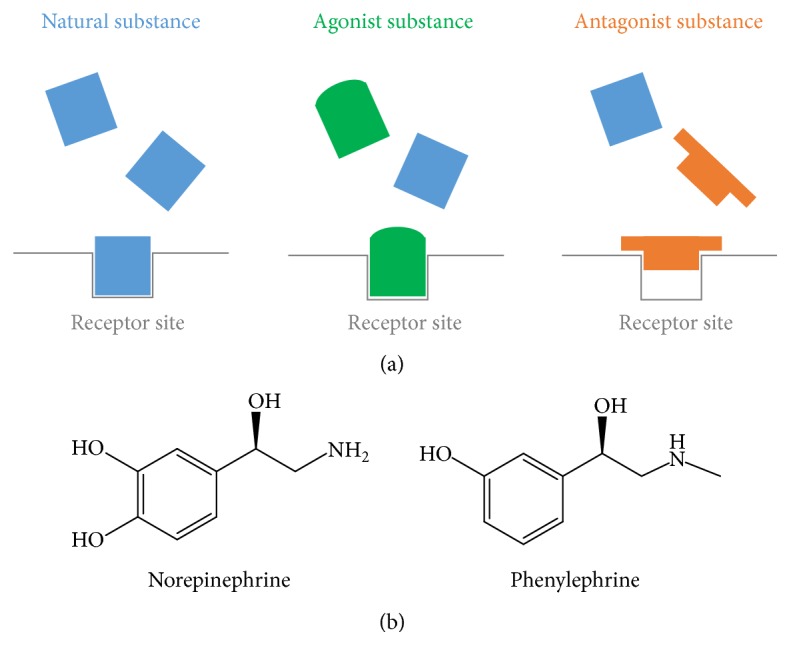
Agonist versus antagonist behaviour. The substances naturally produced by our body act on cellular receptors. The agonist is not the natural substance, but it acts similarly occupying the receptors and activating them, imitating or even enhancing therefore the effect produced by the natural substance. On the contrary, an antagonist mimics the natural substance to take its place in the receptor, blocking thus the cellular activity.

To understand the effect and the mechanism of action of PHCl, it could be useful to state that adrenoceptors are a class of G protein-coupled receptors that are targets of the catecholamines, especially norepinephrine and epinephrine, also known as noradrenaline and adrenaline, respectively. Many cells possess these receptors, and the binding of a catecholamine to the receptor will generally stimulate the sympathetic nervous system. On the other hand, an agonist is a chemical compound capable of simulating the effect of a natural substance produced by our own body. The substances naturally produced by our body act on cellular receptors. The agonist is not the original substance produced by our body, but it acts similarly occupying the receptors and activating them, imitating or even enhancing therefore the effect produced by the natural substance. On the contrary, an antagonist mimics the natural substance to take its place in the receptor. Thus the original substance cannot act as its site is occupied by the antagonist.  Figure 3(a) shows a schematic of the behaviour of agonist and antagonist chemical compounds.

Phenylephrine can be synthetically manufactured, and it is usually found in hydrochloride form as PHCl, which is an acid salt resulting from the reaction of hydrochloric acid with phenylephrine. Converting insoluble phenylephrine into hydrochloride is a common way to make it water-soluble, which allows for a quick absorption into the bloodstream. Phenylephrine has both chemical and pharmacological similarities to norepinephrine [32]. The skeletal formulae of both chemical compounds are shown in Figure 3(b).

A characteristic quality of phenylephrine is the distinctly expressed selectivity to *α*-adrenoceptors. Therefore, as PHCl is primarily *α*-adrenoceptors agonist, it should have little influence on the *β*-adrenoceptors in ciliary muscle. In other words, a drug like PHCl that is a pure *α*-adrenoceptors agonist would be expected to produce pupil dilation with little or no influence in accommodation [21]. But it is, however, possible that PHCl may alter ciliary muscle size or function via *α*-adrenoceptors [17].

AK-Dilate®, Altafrin®, Cyclomydril®, Mydfrin®, Neofrin®, Ocu-Phrin®, and Prefrin® are some of the available brand names prepared as sterile topical ophthalmic solutions containing PHCl.

## 5. Resting State of Accommodation

In an emmetropic eye, the resting state of accommodation (RSA) is usually at an intermediate distance of about one meter and not at infinity [21]. This state can be induced either by being in total darkness or by viewing an empty field under uniform illumination. Therefore, in a normal person at rest, the ciliary muscle exerts 1 D of accommodation. Dual innervation of the ciliary muscle with a balanced tone might be involved in this myopic shift. Therefore, both parasympathetic and sympathetic input to the ciliary muscle are important with regard to determining an individual's RSA [20]. As for the RSA the magnitude of parasympathetic innervation is small (as the accommodation response is around 1 D), and, taking into account that sympathetic innervation operates in only a proportion of individuals and as a function of the concurrent level of parasympathetic activity, the sympathetic input may be limited.

The RSA is referred to as “tonic accommodation,” although the term “dark focus” is also used. If an object is beyond the RSA, effort to relax accommodation would need to be exerted. Contrarily, if an object is nearer than the RSA, an effort to accommodate must be made.

Previous research has demonstrated that the AR is dependent on subject and target characteristics such as pupil size [33, 34], age [35], luminance [36], and spatial frequency [37, 38]. As already stated, in the absence of adequate visual stimuli accommodation adopts an intermediate resting position [39, 40].

## 6. Effects of Phenylephrine Hydrochloride (PHCl) on Accommodation

This section includes the main conclusions that can be outlined from the main research studies that have been performed to try to elucidate the possible effects of PHCl on the accommodation abilities of the human vision system. This literature summary is presented in an essentially chronological order, with the only aim of facilitating comprehension and understanding of the controversy caused in this field throughout the last years.

All the main results that arise from the research reports that have been reviewed are summarized in Table 1. The main outcomes of each report are compiled in a different row in this table, including

- the first author of the report and the year in which it was published,
- the number of patients included in the study and their age range,
- the number of drops of PHCl instilled in each patient and its concentration in %,
- a summary of the measurement procedure to measure the AR,
- the observed effect after PHCl instillation, as it is outlined by the authors of the report.

When reviewing the literature in the field, it is important to consider the PHCl concentration and the number of drops and the instillation protocol (i.e., time between drops and time from last drop to starting the experiment), as all of them are able to influence the obtained results.

While reading this section, the reader is kindly referred to each row of Table 1 to review the main conclusions of each report that varies in the measurement procedure and conclusions outlined.

Biggs and colleagues [6] were the first to report that eight drops of 10% solution of PHCl, instilled intensively at a rate of one drop every two minutes, reduced the amplitude of accommodation (AA) by 0.66 D on average. They employed two different procedures to study the changes in accommodation by this drug. The first was based on the use of a stigmatoscope, which presented a single spot of light viewed on an ophthalmic letter chart. By placing lenses before the eye and requiring the observer to resolve the details of the letter chart while adjusting the stigmatoscope to its sharpest focus, they were able to measure the AR to a variety of accommodative stimuli. The second procedure was based on the use of a subjective Badal optometer to measure the near point of accommodation (NPA). This system has the advantage that changes in position of the target have no effect at all upon either its brightness or its apparent size. In both procedures, a 2.0 mm artificial pupil was used. The effects of the drug on the accommodation were noted 30 minutes after instillation began and had disappeared in two hours. They concluded that intensive topical administration of PHCl caused only a slight recession of the near point, while when the drops were administered once every 15 minutes, it was difficult to demonstrate any effect on accommodation.

Leibowitz and Owens [7] measured, before and after administration of two drops of 10% PHCl, the AR and the dark focus of accommodation with a laser optometer that incorporated the Badal principle. In this technique, a speckle pattern, produced by reflecting the diverged beam of a low-output laser from the surface of a slowly rotating drum, is superimposed in the observer's visual field by means of a beam splitter. If the eye is overaccommodated with respect to the optical distance of the drum's axis, the speckle pattern appears to move in the same direction as the near surface of the drum; if under-accommodated, it appears to move in a direction opposite to the near surface of the drum. When the axis is at an optical distance conjugated to the retina, the speckle pattern appears stationary. They included seven subjects aged 18 to 34: four myopes, two hyperopes, and one emmetrope, all of them wearing their normal optical corrections. The measurements of the AR and the dark focus of accommodation were repeated every 15 minutes for three hours following administration of the drug. The subjects were instructed to fixate carefully a defined target on an outdoor scene for measuring the far point of accommodation (FPA) and to relax their eyes for measuring the dark condition. Pupil dilation was observed 25 minutes after introduction of the drug. Therefore, changes of accommodation due to depth of field or spherical aberration would be expected to occur at this time. Nevertheless, no systematic change of either the far point of accommodation or the dark focus was evident in the results.

Eight years later, Garner and coworkers [8] also measured the accommodative state with a laser optometer based on the Badal principle through the analysis of moving speckle patterns. They used a 6/9 Snellen letter with a background luminance of 3 cd/m^2^ to measure the FPA. The NPA was determined as the closest distance to which a line target could be moved towards the eye without noticeable blur. And the RSA was determined with the laser optometer after 5 minutes in complete darkness. Two drops of PHCl 10% were instilled in each eye. Three subjects were included in the study, and the FPA, NPA, and RPA were determined at approximately hourly intervals for the duration of each trial. The drug treatment produced mydriasis and reduced the NPA in all subjects by 2.5–3.0 D under natural pupils. A typical accommodative lag of 1 D was exhibited by all subjects. The RPA varied between 2.0 and 2.5 D. The FPA and RPA showed no significant variations during the course of changes in pupil diameters.

Zetterström [9] studied the influence of different concentrations of PHCl on the accommodation abilities of 10 healthy volunteers ranging in age from 22 to 29 years. Lenses were placed in front of the eye corresponding to the refraction for far distance and with the addition of a −3.0 D lens to move the near point to a point which allowed a proper distance per dioptric unit in the determination of accommodation. All measurements were made using a 2 mm artificial pupil. The test subject looked at the smallest optotype on a RAF near-point rule (Clement Clarke Ltd.), beginning from a distance of half a meter and then slowly moving the text towards the eye until the text became blurred. The determination was performed by the examiner, and the mean value of three determinations was calculated. All volunteers were tested without the instillation of PHCl and after the instillation of one drop of PHCl 0.1%, one drop of PHCl 1%, and one drop of PHCl 10% with a wash-out of at least 2 days between experiments. As a result, the mean values of accommodation in diopters showed a fairly good correlation between dose and decrease in accommodation. Statistical analysis revealed that there were significant differences in the ability to accommodate between the three different solutions. Nevertheless, great individual differences were found. In particular, two out of the 10 subjects were extremely sensitive. For them, the accommodation was reduced by 5.6 and 8.8 D after one drop of 10% PHCl. The author concluded that PHCl had an effect on accommodation and that this effect could amount to 2 or 3 D.

Mordi and colleagues [10] analyzed the mydriatic and cycloplegic effects of either cyclopentolate 0.1% or PHCl 10% over a period of up to 6 hours after drug instillation. They included five subjects aged between 25 and 43 years. Two drops of PHCl were instilled separated by 2 minutes, and the monocular static and dynamic AR were assessed with an infrared optometer. High-contrast targets were employed with target-eye vergence of −5.0, −4.0, −3.0, −2.0, and −1.0 D. A supplementary lens allowed an additional effective vergence of 0.0 D to be presented. Target size was scaled so that the smallest detail of each target subtended the same visual angle at all object distances. The authors characterized the dynamic change in response to an abrupt change in target vergence in terms of two temporal parameters, the reaction time and the response time. The reaction time is defined as the time elapsing between the stimulus change and the start of the corresponding response, and the response time is the time taken to complete the response. The reaction times were little influenced by the drug used. Nevertheless, both drugs increased the response time, with cyclopentolate having the larger effect. Besides, considerable intersubject variations in response characteristics were observed particularly with respect to the initial direction of response. Regarding the effects of the drugs upon the steady state response levels, both drugs had considerable effects, and these effects varied as a function of time. With 10% PHCl, the magnitude of the slope of the response/stimulus curve was reduced. According to the authors, this slope reduction was obviously closely related to the recession of the near point found by Garner et al. [8] and the reduction in the AA found by Zetterström [9] with the same drug. The authors concluded that neither drug can be considered to be an “ideal” mydriatic, since each produces cycloplegic effects, including a slowing of the dynamics of the AR, a recession of the near point, and a reduction in the slope of the AR/stimulus curve, which have approximately the same temporal characteristics as the mydriatic effects.

In an additional report, Mordi and coworkers [11] stated the conclusions of a different study to analyze the effect of the topical instillation of PHCl on accommodation. Factors considered in this study were iris colour and the influence of the prior application of a topical anesthetic (proparacaine hydrochloride) before PHCl instillation. Baseline measurements of AA, refractive status, and pupil diameter were obtained before any drops were instilled in the eyes of 10 Caucasian subjects all within the age range of 20 to 26 years. Each subject received 1 drop of topical anesthetic in 1 eye. Three minutes later the subjects were instilled with 1 drop of PHCl 2.5% in each eye. The AA was measured while the subject wore the appropriate optical correction for his or her distance refractive error. Donders' push-up method [41] was used to measure the amplitude while the subject read the words in the 0.62 M test target at 40 cm. Amplitude measurements were repeated every 5 min until the subject's minimum value was reached, and then the measurements were taken every 20 min until 50% recovery of the amplitude occurred. Pupil size was measured under the same lighting conditions. The authors obtained that PHCl 2.5% does inhibit accommodation by about 15% when used alone and by over 25% when preceded by anesthetic instillations. Besides, anesthetizing the cornea improves the ability of PHCl to dilate the pupil. The authors concluded that prior application of an anesthetic prolongs both the depression effect of PHCl on the accommodation and the mydriatic effect, in eyes with either lightly or more heavily pigmented irides.

Two years later, the possible influence of PHCl in the activity of the ciliary muscle was studied using in vitro samples. Zetterström and collaborator [12] studied the in vitro pharmacological characteristics of adrenoceptors of the human ciliary muscle. They used human tissue obtained from 30 eyes which had been enucleated 6–24 hr after death and used previously for corneal transplantations. By means of attaching strips of the meridional and circular portion of the ciliary muscle to a tension gauge in an organ bath they were able to monitor isometrically the effect of drugs added to the perfusion medium. As a result, ciliary muscle from only three out of eight eyes relaxed in responses to PHCl.

Later on, Gimpel et al. [13] performed a large sample study over a period of 5 years to investigate the effects of PHCl 2.5% on the subjective AA in 160 healthy eyes without the prior use of a topical anesthetic. The NPA was measured monocularly by Donders' push-up method: the best acuity target was slowly moved towards the test eye until it was blurred; then the distance of the target from the eye and estimated spectacle plane was converted into dioptric equivalents. All measurements were assessed through the natural pupil under ambient illumination of around 150 lux. After the instillation of a single drop of PHCl 2.5% into the test eye, measurements were repeated for the test eye and contralateral eye every 5 min for 30 min and then every 10 min until a total time period of 90 min. By 30 min, a net decrease in the subjectively assessed average AA of 1.22 D occurred, that is, a net reduction of 10.9% compared to the initial baseline readings. At 60 min, a reduction of 1.39 D was recorded, although large standard deviations about the mean values were obtained, reflecting the fact that some subjects showed a reduction in amplitude, and some showed no change, while a few showed an increase in amplitude. This study clearly confirmed that PHCl could reduce the AA as assessed subjectively.

Four years later, Eyeson-Annan and colleagues [14] performed a study to identify any significant difference between maximum pupil dilation and accommodation after PHCl 10% alone and PHCl 10% combined with tropicamide 1%. In the first dilation regimen, one drop of 0.4% oxybuprocaine hydrochloride was instilled into the right eye and then the left eye of each subject, followed 30 seconds later by one drop of PHCl 10% and 5 minutes later by a second drop and a third drop 5 minutes later. Oxybuprocaine hydrochloric acid was instilled into the subjects' eyes to improve absorption of the PHCl. In the second dilation regimen, one drop of 0.4% oxybuprocaine hydrochloride was instilled into the right eye and then the left eye of each subject, followed 30 seconds later by one drop of PHCl 10% and followed 30 seconds later by one drop of tropicamide 1%, repeating the instillation of these two chemical agents 5 and 10 minutes later. The authors tested also a reversal regimen to counteract the effects of the mydriasis, by instilling one drop of 0.4% oxybuprocaine hydrochloride into the right eye and then the left eye of each subject, followed 30 seconds later by a drop of thymoxamine hydrochloride 0.5%. A total of 47 subjects participated in the study. The subjects' AA was measured binocularly using a near-reading chart at the 0.7 line. Two accommodation levels were measured: (1) the reading distance and (2) the near-chart distance at which the letters began to blur. Those subjects who wore glasses were instructed to use them for all of the tests. Accommodation was measured using the near-reading chart just before dilation, 20 and 40 min after instillation of the dilating drops. Reversal regimen was instilled 40 min after instillation of the dilating drops, and measurements were taken again 40 min after the instillation of the reversal drops. The authors concluded that accommodation was impaired significantly more after PHCL and tropicamide than after PHCl alone. Thus, PHCl alone had no significant effect on the AA of the subjects, reporting mean values of 1.60 D and 1.57 D at 20 min and 40 min after instillation of dilating drops, respectively, in comparison with a baseline value of 1.53 D. However, tropicamide in combination with PHCl had significantly decreased the mean AA of the subjects, obtaining values of 0.70 D and 0.80 D at 20 min and 40 min after dilating drops. Measuring 40 minutes after reversal with thymoxamine resulted in a mean accommodation value of 1.12 D, which still was significantly lower than the baseline value.

Next report was presented by Culhane and coworkers [2], who made a study to identify the action of *α*-adrenoceptors on the closed-loop AR. Ten visually normal subjects participated in the study, in which monocular temporal AR were measured objectively using a continuously recording dynamic tracking infrared optometer. They used a visual Badal stimulus deflector system to change optically the vergence (apparent distance) of the accommodation stimulus, which allowed stimulus vergence to be modulated without changing stimulus size, position, or luminance. A 4-mm artificial pupil was imaged in the subject's natural entrance pupil to remove any variation in performance caused by changes in depth of focus due to pupil dilation. The dynamics of the AR to sinusoidal (0.05, 0.1, 0.2, 0.3, 0.4, and 0.6 Hz) and stepwise (0.05 Hz) modulations in target vergence over a 2- to 4-D range were investigated before and after (approximately 55 minutes) the instillation of 1 drop of PHCl 2.5% into the lower fornix. The authors obtained a significant increase in accommodative gain at low- and mid-temporal frequencies, although no significant difference in phase lag was detected. These results showed that additional stimulation of the *α*-inhibitory receptors with PHCl 2.5% allowed the accommodative system to track more accurately at low- and mid-temporal frequencies, optimizing then the AR. According to the authors, as an optimum balance between the parasympathetic and sympathetic control of accommodation is necessary to respond to temporal variations in target distance and to prevent adaptation after sustained near vision, these results suggested that the sympathetic component of the response is necessary to provide maximum negative accommodation.

In 2002, Do and coworkers [15] measured subjectively the AA with the push-up technique and objectively with the Hartinger Coincidence Refractometer in a set of 10 subjects. They also assessed accommodative dynamics with an infrared optometer (PowerRefractor). Measurements were taken before and at 15-minute intervals for 90 minutes after instillation of PHCl 2.5%. In this study, all subjects showed a decrease in AA measured with the subjective push-up technique (mean 17%, max decrease at 30–90 minutes) after instillation of PHCl. Nevertheless, AA measured objectively with the Hartinger Coincidence Refractometer showed no decrease in maximum AA. According to the authors, this is likely to occur due to mydriasis of the pupil and decreased depth of focus rather than sympathetic inhibition of the ciliary muscle. Regarding dynamic measurements, some subjects showed a decrease in peak velocity of accommodation, suggesting that PHCl should not be used to dilate the pupil when testing accommodative dynamics.

The effects of PHCl 10% on pupil diameter and accommodation were then studied in rhesus monkeys [5], as they represent a unique model for human accommodation because they have high AA and an accommodative mechanism and anatomy similar to that of humans. The purpose of this study, conducted by Ostrin and Glasser, was to determine whether PHCl affects Edinger-Westphal- (EW-) stimulated accommodation in rhesus monkeys. Static and dynamic EW-stimulated AR were studied in five iridectomized rhesus monkeys before and after phenylephrine instillation. An electrode implanted in the EW nucleus of the monkeys' midbrain was used to stimulate the open-loop accommodation. This method allows for an AR that is not affected by pupil size or visual feedback and that can be rigorously controlled by stimulus amplitude, differentiating so effects of PHCl on the ciliary muscle versus effects due to secondary optical factors resulting from mydriasis. A Hartinger Coincidence Refractometer was used to measure the AA, and infrared photorefraction was used to assess the dynamic AR. After the baseline recordings, two doses of 0.1 mL PHCl 10% were instilled topically, separated by 2 minutes. The effects of PHCl on dynamic accommodation were established in terms of peak velocities of accommodation and disaccommodation. The authors obtained post-PHCl AA similar to pre-PHCl amplitudes, whereas dynamic analysis of the AR showed linear peak velocity versus AA relationships that were not statistically different before and after PHCl. The authors of this study concluded that although there are individual differences before and after the instillation of PHCl, these differences are not systematic, and within the resolution of the methods there are no significant effects of PHCl on AA, dynamics, or RSA. Therefore, adrenergic stimulation causes strong pupil dilation in noniridectomized monkey eyes but does not affect EW-stimulated AA or dynamics in anesthetized, iridectomized rhesus monkeys.

Recently, in 2012, Sarkar et al. [16] performed a study to determine the combined impact of PHCl concentration and pupil size on the static and dynamic characteristics of accommodation of sixteen visually normal adults. The study had four different conditions regarding the PHCl concentration (without PHCl and with PHCl 2.5%, PHCl 5%, and PHCl 10%) and five viewing conditions (viewing with natural pupils and with 8, 6, 4, and 1 mm diameter artificial pupils). A total of three drops were instilled, one drop every 15 min, and the experiment started 1 hour after instillation of the first drop. Subjects watched a high-contrast, high spatial frequency visual target displayed on one of the two liquid-crystal display screens that were placed at 67 and 33 cm, respectively, in front of the subject. The visual target was electronically switched between the two screens, once every 4 s, thereby creating an accommodative demand of 1.5 D. The viewing was monocular while AR was recorded bilaterally. They measured a peak velocity of accommodation with no PHCl significantly larger than those with all three concentrations of PHCl, while the data for the three drug concentrations were not significantly different from each other. Overall, their results indicated that PHCl had a small but statistically significant negative impact on the response magnitude and peak velocity of accommodation but not that of disaccommodation. There appeared to be no obvious interaction between drug concentration and pupil size on accommodative performance, suggesting that the pharmacological effect of PHCl and the optical effect of increased pupil diameter following PHCl instillation both contribute towards the reduction in accommodative performance. According to the authors, the reduction in accommodative performance is modest and does not carry a large clinical significance, meaning that PHCl could therefore be used to achieve pupil mydriasis without dramatically hampering accommodation.

In the same year Richdale et al. [17] conducted the first study to examine the effect of PHCl on the human ciliary muscle in vivo. They aimed to determine if, in response to topical administration of 2.5% PHCl, there were changes in (1) the dimensions of the ciliary muscle and (2) the accommodative contraction of the ciliary muscle. The Visante Anterior Segment OCT (Carl Zeiss Meditec, USA) was used to image the ciliary muscle in relaxed and accommodated states (0 and 4 D targets) in a set of 25 subjects. Pupil size and accommodative function were also measured. AR at 0 and 4 D stimulus levels was measured subjectively and objectively. For subjective testing, maximum monocular accommodation was determined using the push-up to blur technique and a 1 mm letter target. Objective static AR was measured with the Grand Seiko WV 500 Auto-Refractor (Grand Seiko Ltd., Japan) and a 2 mm letter target on a Badal lens track. Measurements were repeated 30 minutes after topical administration of one drop of 1% proparacaine and one drop of PHCl 2.5%. Proparacaine was used both to increase patient comfort and because application of proparacaine has been shown to increase the effect of PHCl, especially in patients with dark irides [11]. They obtained that the maximum subjective AR was reduced by about 1 D following instillation of PHCl, although the drug did not affect the objectively measured AR. Besides, the cross-sectional ciliary muscle dimensions and contractility to a 4 D stimulus were not altered by the instillation of PHCl 2.5%. This finding suggests, according to the authors, that there is no physiological effect on the accommodative system due to the effect of PHCl.

The last contribution to the field was dated 2016. Bernal-Molina and colleagues [18] tested the hypothesis that changes in accommodation after PHCl instillation are due to changes in optics and not to changes in the function of the ciliary muscle. They studied the effect of PHCl both on static and on dynamic accommodation. The effect on static accommodation was assessed for 8 eyes by computing the stimulus-response curve from the wavefront obtained at different vergence. Measurements were taken with the IRX3 commercial aberrometer (Imagine Eyes, France) for natural pupils before and after instillation of 2 drops of PHCl 10%. From these curves, the authors obtained the objective AA and the AR up to 6 D. The effect of phenylephrine on dynamic accommodation was assessed for 6 eyes by measuring the AR at 10 Hz to a sinusoidally moving stimulus seen through a 3-mm artificial pupil. The stimulus moved between 1 D and 3 D from the subject's far point at 0.2 Hz. Measurements were taken with a custom-made optical system, and the authors computed the gain and phase of the dynamic responses. AR was calculated taking into account the effects of higher-order aberrations (minimum RMS refraction) and without them (paraxial refraction). The authors obtained that, for static accommodation with the minimum RMS calculation of AR, the mean AA (and 95% confidence interval) changed after PHCl instillation by 0.51 D (0.14 D, 0.88 D). The change of the AR at a 6 D of accommodative demand was 1.00 D (0.64 D, 1.36 D). However, the values obtained for paraxial AR were −0.20 D (−0.55 D, 0.15 D) and 0.22 D (−0.20 D, 0.64 D), respectively. For dynamic accommodation, the authors found a mean difference in the AR gain of 0.09 D (−0.10 D, 0.19 D) with minimum RMS and −0.08 D (−0.14 D, 0.02 D) for paraxial refraction. The authors concluded that whereas they found a clear, statistically significant decrease in AR after the instillation of PHCl, they did not find any effect on static and dynamic accommodation when higher-order aberrations were not taken into account in the calculation of AR. According to the authors, as paraxial refraction depends mainly on the ciliary muscle, its function seems to be unaffected by PHCl according to their results. Therefore, differences in reported effects of PHCl may be due to the methods used to calculate the AR.

## 7. Conclusions

First investigations suggested some loss of functional accommodation in the human eye after PHCl instillation. Subsequent research studies, based on different measurement procedures, obtained contradictory conclusions, causing therefore an unexpected controversy that has been spread almost to the present days.

This manuscript reviews the main research studies that have analyzed the effect of PHCl on the accommodative system of the human eye and summarizes their main conclusions, describing the different measurement procedures that have been used in each one.

As a brief summary, it is worth noting that AR measurements after PHCl instillation performed by subjective methods such as Donders' push-up technique obtained significant reductions in the AA of up to several diopters. Nevertheless, the experiments based on objective measurement methods, such as the study of the ciliary muscle size by OCT images or the use of an electrode implanted in monkeys' midbrain to stimulate accommodation without the feedback giving by the pupil size, conclude that there are no significant effects due to PHCl on AA, dynamics, or RSA.

All in all, it seems that the most accepted conclusion is that subjective measurements are affected by mydriasis produced by PHCl instillation, in the sense that the alterations measured on accommodation are likely to occur due to optical changes in the eye following pupil dilation. The iris dilates after PHCl, reducing the depth of field and affecting thus the subjects' perception of blur. On the contrary, objective methods, which are able to separate accommodation measurements from the optical effects of the pupil dilation on the perception of the image, conclude that PHCl instillation has no effect over the accommodative abilities of the human eye.

## Figures and Tables

**Table 1:** Summary of the measurements procedures and the observed effects after PHCl instillation for the main reports that have been reviewed. The asterisks denote the studies in which the authors found some effects over the accommodative abilities of the human eye after PHCl instillation (these studies are normally linked to a high level of accommodation response, i.e., high parasympathetic response).

First author (year) (Ref)	Subjects (Age range)	PHCl drops (% concentration)	Measurement procedure	Observed effect after PHCl instillation
Biggs (1959) [6]^*∗*^	5 (n/a)	8 (10%)	Subjective Badal optometer to measure the near point of accommodation (2 mm artificial pupil).	Recession of the near-point averaging 0.66 D.
Leibowitz (1975) [7]	7 (18–34)	2 (10%)	Laser optometer with Badal principle and moving speckle patterns.	No change of either the far point of accommodation or the dark focus was evident in the results.
Garner (1983) [8]^*∗*^	3 (n/a)	2 (10%)	Laser optometer with Badal principle and moving speckle patterns. FPA was measured using a 6/9 Snellen letter. NPA was the closest distance to which a line target could be moved towards the eye without noticeable blur. RPA was determined with the laser optometer after 5 minutes in complete darkness.	Reduction of the NPA in all subjects by 2.5–3.0 D under natural pupils. A typical accommodative lag of 1 D was exhibited by all subjects. The RPA varied between 2.0–2.5 D.
Zetterström (1984) [9]^*∗*^	10 (22–29)	1 (0.1%, 1%, 10%)	The subject looked at the smallest optotype on a RAF near-point rule, beginning from a distance of 50 cm and then slowly moving the text towards the eye until blurring (2 mm artificial pupil).	There were significant differences in the ability to accommodate between the three different solutions. Although great individual differences were found, PHCl has an effect on accommodation that can amount to 2 or 3 D.
Mordi (1986) [10]^*∗*^	5 (25–43)	2 (10%)	Monocular static and dynamic AR were assessed with an infrared optometer. Reaction time and response time were used to characterize the dynamic accommodation.	Cycloplegic effects, including a slowing of the dynamics of the AR, a recession of the NPA and a reduction in the slope of the AR/stimulus curve (50% approx. at two hours after instillation).
Mordi (1986) [11]^*∗*^	10 (20–26)	1 (2.5%)	Donders' push-up method was used to measure the AA while the subject read the words in the 0.62 M test target at 40 cm.	Accommodation inhibition by about 15% when used alone and by over 25% when preceded by anesthetic instillations.
Zetterström (1988) [12]	8 (34–90)	40 mL organ bath (dose responses: 5·10^−6^–5·10^−3^ M)	In vitro pharmacological test using human ciliary muscle tissue. Muscle strips of the meridional and circular portion of the ciliary muscle were attached to a tension gauge in an organ bath. Drug was added to the perfusion medium.	Ciliary muscle from only 3 out of 8 eyes relaxed in response to the drug.
Gimpel (1994) [13]^*∗*^	160 (20–30)	1 (2.5%)	NPA was measured monocularly by Donders' push-up method. All measurements were assessed through the natural pupil.	At 30 min a net decrease in the subjectively assessed average AA of 1.22 D was obtained. At 60 min, a mean reduction of 1.39 D was recorded.
Eyeson-Annan (1998) [14]	47 (20–79)	3 (10%)	AA was measured binocularly using a near-reading chart at the 0.7 line. Two accommodation levels were measured: (1) the reading distance and (2) the near-chart distance at which the letters began to blur.	PHCl alone had no significant effect on the AA of the subjects, reporting mean values of 1.60 D and 1.57 D at 20 min and 40 min after instillation of dilating drops, respectively, in comparison with a baseline value of 1.53 D.
Culhane (1999) [2]	10 (20–28)	1 (2.5%)	Monocular dynamic AR were measured objectively, in target vergence over a 2- to 4-D range, using a continuously recording infrared optometer (4-mm artificial pupil).	PHCl allowed the accommodative system to track more accurately at low- and mid-temporal frequencies, optimizing then the AR.
Do (2002) [15]^*∗*^	10 (24–28)	n/a (2.5%)	Accommodative amplitude was measured subjectively with the push-up technique and objectively with the Hartinger Coincidence Refractometer.	All subjects showed a mean decrease of 17% in accommodative amplitude measured with the subjective push-up technique. Accommodative amplitude measured objectively with the Hartinger Coincidence Refractometer showed no decrease.
Ostrin (2004) [5]	5 anesthetized rhesus monkeys	0.2 mL (10%)	An electrode implanted in the monkeys' midbrain was used to stimulate accommodation (this allows for a response that is not affected by pupil size or visual feedback). A Hartinger Coincidence Refractometer measured the AA, and infrared photorefraction was used to assess dynamic responses.	Although there were individual differences before and after the instillation of PHCl, these differences were not systematic, and within the resolution of the methods there were no significant effects of PHCl on accommodative amplitude, dynamics, or resting position.
Sarkar (2012) [16]^*∗*^	16 (21–30)	3 (2.5%, 5%, 10%)	Subjects watched a target displayed on liquid-crystal screens that were placed at 67 and 33 cm. The visual target was electronically switched between the two screens, once every 4 s, thereby creating an accommodative demand of 1.5 D (natural pupil and 8, 6, 4, 1 mm artificial pupils).	PHCl had a small but statistically significant negative impact on the response magnitude and peak velocity of accommodation but not that of disaccommodation. The reduction in accommodative performance is modest and does not carry a large clinical significance.
Richdale (2012) [17]	25 (18–40)	1 (2.5%)	Subjective measurements were obtained using the push-up to blur technique. Objective static AR was measured with an autorefractor. OCT was used to in vivo image the ciliary muscle in relaxed and accommodated states.	Maximum subjective AR was reduced by about 1 D. The drug did not affect the objectively measured AR. Muscle dimensions and contractility to a 4 D stimulus were not altered by the instillation of PHCl.
Bernal-Molina (2016) [18]	8 (n/a)	2 (10%)	Static accommodation (AA and AR) was assessed by a commercial aberrometer computing the stimulus-response curve from the wavefront obtained at different vergence. Dynamic accommodation was also assessed by measuring the AR at 10 Hz to a sinusoidally moving stimulus (3-mm artificial pupil).	Any effect was measured on static or dynamic accommodation when higher-order aberrations were not taken into account in the calculation of AR. As paraxial refraction depends mainly on the ciliary muscle, its function seems to be unaffected by PHCl.

AA: amplitude of accommodation; AR: accommodative response; D: diopters; FPA: far point of accommodation; min: minutes; n/a: not available; NPA: near point of accommodation; OCT: Optical Coherence Tomography; PHCl: Phenylephrine hydrochloride; RPA: resting point of accommodation.
